# Some Important Facts Regarding Cholera

**Published:** 1854-03

**Authors:** John Furlonge

**Affiliations:** St. John’s, Antigua


*Some Important Facts regarding Cholera.* By JOHN FURLONGE, M. D. *Antigua.*—The following facts are so interesting that I hasten to forward them for insertion in your widely-circulated journal. The ship *Glenmenna,* from Liverpool, bound to New Orleans, with 500 emigrants, put into the Port of Charleston, Nevis, for medicines and provisions. She had lost between twenty and thirty emigrants from cholera since she left England, and had then between twenty and thirty lying ill with the disease. The authorities at Nevis prohibited communication with the shore, and the President sent his secretary alongside, who boarded her, to warn the captain to that effect. The ship was supplied, however, with what she wanted. She lay at anchor very near to the shore, and remained *sixteen* hours, and was reported by the persons who went to her as very dirty, and disgustingly and intolerably offensive. The ship arrived on the 22d of November, and on the 4th of December (twelve days after) malignant cholera of a terribly fatal nature broke out at Charleston. Amongst the first who suffered was the secretary, who went on board; he recovered. The first five cases, a father and four children, all died, and in fourteen days it swept off upwards of 100, ten being the average daily mortality. On Sunday last, the 18th instant, thirty are reported to have died, and on the next day twenty-eight.—We here, at Antigua, are very much alarmed, as Nevis is about fifty miles west, and the wind for some time had prevailed from that quarter. The profession here are almost to a man non-contagionists, but this "blow" has, in some degree, shaken our doctrine, and the Board of Health has ordered strict quarantine between the islands. This outbreak at Nevis—one of the healthiest West India islands, isolated, and which escaped yellow fever, so lately prevalent in the neighboring colonies —would almost seem to settle the question of contagion, and to afford a kind of *experimentum crucis* on the subject. We are doing in Antigua all we can to avert the pest from our shores, so far as sanitary measures can be effectual. I may mention that it is reported that several of the ship's men got on shore at Nevis, and became very drunk; but this requires authentication.* The disease is reported to have attacked chiefly the poorer classes, and the persons about butchers' shambles are said to have suffered much, and all filthy localities. The town is represented as in a very bad condition as regards sanitary requirements, and the sea-shore (the town is close to it) is made the receptacle for *all* the filth, domestic and otherwise, and is intolerably disgusting.—*The Lancet.*

*St. John's, Antigua, December*, 1853.

*I have since heard that this is true, and that five dead bodies were thrown overboard in the roadstead, and it was at first believed that the illness arose from eating fish which had preyed on the bodies. The people will not touch fish.

